# Transgenic solutions to increase yield and stability in wheat: shining hope or flash in the pan?

**DOI:** 10.1093/jxb/erz077

**Published:** 2019-03-11

**Authors:** José L Araus, Maria D Serret, Marta S Lopes

**Affiliations:** 1 Integrative Crop Ecophysiology Group, Plant Physiology Section, Faculty of Biology, University of Barcelona, Barcelona, and AGROTECNIO Center, Lleida, Spain; 2 Sustainable Field Crops Program, Institute for Food and Agricultural Research and Technology (IRTA), Lleida, Spain; 3 The International Maize and Wheat Improvement Center (CIMMYT), Ankara, Turkey

**Keywords:** Drought, *HaHB4*, transgenics, wheat, yield

## Abstract

This article comments on:

**González FG, Capella M, Ribichich KF, Curín F, Giacomelli JI, Ayala F, Watson G, Otegui ME, Chan RL.** 2019. Field-grown transgenic wheat expressing the sunflower gene *HaHB4* significantly outyields the wild type. Journal of Experimental Botany 70, 1669–1681.

**Second-generation transgenic crops have the potential to transform agriculture, but progress has been limited, and particularly so in wheat where no transgenic cultivar has yet been approved. Taking on the challenge, González *et al.* (2019) report that transgenic wheat lines carrying a mutated version of the sunflower transcription factor (*HaHB4*), belonging to the homeodomain-leucine zipper family (HD-Zip I), had increased yield and water use efficiency across a range of environments, with particular benefits under stress. It is an important step forward in an area where progress is urgently needed, though it is too early to claim that transgenic wheat will form the backbone of a second Green Revolution.**

To meet the growing demand for food, together with the challenges imposed by climate change, substantial improvements in yields of major crops are needed. This includes wheat, where globally the multi-year tendency for growth in yield is decreasing (Passioura, 2012) or even stagnating (Driever *et al.*, 2017). Current and expected future relative rates of progress in yield potential and drought adaptation in wheat are a matter of real concern, and insufficient to meet the projected demand for cereals by 2050 (Hall and Richards, 2013). There are three major challenges: increasing yield potential, protecting yield potential from different types of stress, and increasing resource use efficiency to ensure sustainability (Hawkesford *et al.*, 2013).

## Current scenario urges for ‘miracles’

It is in this context that the idea of conventional breeding losing the battle against climate change is frequently presented as support for a transgenic future; the claim is that the second generation of transgenic crops is projected to mitigate abiotic stress effects (ISAAA, 2017) and produce a quantum leap in productivity (a so-called ‘second Green Revolution’). However, to date expectations placed on transgenic crops have not been realized. So why has the potential of a transgenic road to agriculture’s ‘Promised Land’ not been realized?

There are a number of reasons that explain this lack of success. With complex multi-faceted traits such as drought resistance or yield potential, contributions from genetic transformation have been slight. The general reasons for this bottleneck are outlined elsewhere (Araus *et al.*, 2008; Passioura, 2010; Sinclair, 2011). Most evaluated transgenic lines have failed to translate the benefits observed in controlled environments to field conditions (Passioura, 2012). For example, the search for generic drought resistance using single-gene transformations has been disappointing. Research has typically concentrated on survival of plants suffering from severe water stress, which is rarely an important trait in grain crops such as wheat. Moreover, in many cases survival has simply been explained by reduced plant size and concomitant slower water uptake compared with the wild-type variety (Morran *et al.*, 2011), with the transgenic plants growing in pots or containers instead of the field. This was certainly the case for two members of the wheat homeodomain-leucine zipper I (HD-Zip I) family of transcription factors, which regulate development after plants are exposed to environmental stimuli and stresses. After they were introduced as transgenes into wheat, the plants showed improved resistance to drought and frost, but they exhibited undesirable phenotypic characteristics such as reduced size, biomass and yield (Kovalchuk *et al.*, 2016; Yang *et al.*, 2018).

The implementation of selected genes in breeding programmes requires consideration of specific genotypes as well as agronomic and climate conditions and the fact that many of the genes are members of multigene families (Nadolska-Orczyk *et al.*, 2017). It is in this context that realistic experimental protocols (i.e. field trials) to screen new cultivars are crucial. Indeed, the effects of a particular transgene can sometimes be reversed when plants are moved from the glasshouse to the field (Zeller et al., 2010). In a study conducted at CIMMYT, even though transgenic DREB1A-wheat lines were selected under greenhouse conditions in recovery after severe water stress, under field conditions the same group of transgenic lines did not generally outperform the controls in terms of grain yield (Saint-Pierre *et al.*, 2012). Earlier this decade Passioura (2012) had already identified that from more than 1000 papers about ‘drought tolerant’ transgenics, very few reports evaluated the plants in field conditions. This trend has continued: in papers published from 2010 to the present on transgenic wheat and maize less than 5% included the word ‘field’ in the title.

One of the few notable exceptions and among the earliest that stressed the importance of field evaluation was reported by Castiglioni *et al.* (2008), who indicated some improvement in yield when maize transformed with the bacterial RNA chaperones CspB and CspA experienced drought at flowering. This work generated the first of a still scarce number of commercial transgenic crops resistant to abiotic stresses (DroughtGard™). Another example is the study of González *et al.* (2019) on wheat expressing the transcription factor *HaHB4*, which also belongs to the HD-Zip I family of transcription factors. Its expression is induced by ABA, water deficit, ethylene and jasmonic acid, among other environmental and hormone factors. Moreover, *HaHB4* action is not dependent on the response triggered by either RD19 or DREB1a, traditional candidates related to water deficit responses. Apparently the molecular mechanism triggered by this transcription factor does not involve stomatal closure but is associated with cell membrane stabilization (Cabello and Chan, 2012). Nevertheless, the specific mechanisms at work in wheat are unknown (González *et al.*, 2019). Interestingly, the introduction of *HaHB4* into wheat does not seem to affect grain and forage nutritional characteristics (Ayala *et al.*, 2019).

## Other transgenic avenues in wheat

The increase in yield potential and sustainability in wheat through transgenes involves an ideotypic definition of potential targets for transformation (Box 1). Among other recent transgenic approaches in wheat it is worth mentioning the study of Yadav *et al.* (2015) on heterotrimeric nuclear factors Y (NF-Ys), which are involved in the regulation of various vital functions in all eukaryotic organisms. Under optimal watering conditions, transgenic wheat plants overexpressing *TaNF-YB4* produced significantly more spikes but other yield components did not change. This resulted in a 20–30% increased grain yield compared with untransformed control plants. Under water-limited conditions transgenic lines maintained parity in yield performance (Yadav *et al.*, 2015). However, while the authors claim that analysis of T_2_ plants was performed in large deep containers in conditions close to field trials, the agronomic yield components are clearly lower than those of normal (i.e. conventionally bred) wheat in the field. Besides these considerations, an increase of 20–30% is a very significant improvement considering that the low genetic gains obtained through conventional wheat breeding are around 1% per year (Lopes *et al.*, 2012) or even less (Fischer *et al.*, 2011). This leads to questions about the non-transgenic lines used for comparison, and specifically whether local well-adapted check varieties (obtained from conventional breeding) should be used to establish a clear baseline by which to gauge the actual yield improvement.

Box 1. **Different pathways for increasing yield potential and stress adaptation in wheat that may be modified using transgenics**

Promising approaches target complex traits related to specific requirements for drought resistance at key stages of the crop life cycle: establishment, vegetative development, floral development and grain growth (Passioura, 2012). Examples of productivity-related genes in wheat, with a potential impact on agronomic yield components, have been classified functionally by Nadolska-Orczyk *et al.* (2017) into several groups, and these may serve as targets for transgenics. These include (1) transcription factors, regulating spike development, which mainly affect grain number; (2) genes involved in metabolism or signaling of growth regulators – cytokinins, gibberellins, and brassinosteroids – which control plant architecture and consequently stem hardiness and grain yield; (3) genes determining cell division and proliferation mainly impacting grain size; (4) floral regulators influencing inflorescence architecture and consequently seed number; and (5) genes involved in carbohydrate metabolism having an impact on plant architecture and grain yield. In addition, modulated expression of flowering genes, which regulate photoperiod and vernalization-dependent floral induction, might be advantageous for spring or winter varieties (Nadolska-Orczyk *et al.*, 2017). Moreover, increasing photosynthetic rates of laminar and non-laminar organs and the capacity to access and use larger amounts of resources (such as water or nutrients) are also functional targets for transgenesis (e.g. Sivamani *et al.*, 2000; Kulkarni *et al.*, 2017). Sw, sowing; Em, emergence; FI, floral initiation; DR, double ridge; TS, terminal spikelet; Hd, heading; At, anthesis; GF, grain filling; PM, physiological maturity.

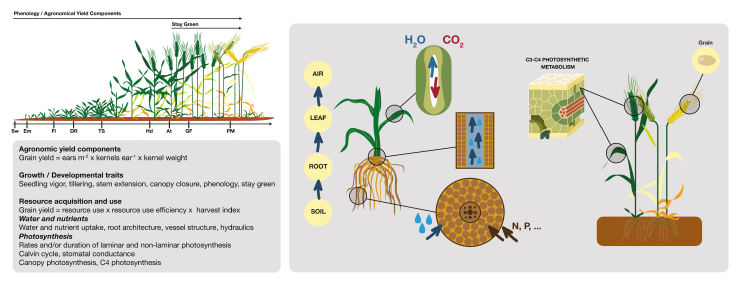

Regulation of root architecture is essential for maintaining plant growth under adverse environmental conditions. Overexpression of a nitrate-inducible NAM, ATAF and CUC (NAC) transcription factor in wheat has been reported to enhance root growth and the ability to acquire nitrogen, hence increased grain yield and nitrogen accumulation (He *et al.*, 2015). While the advantage in grain yield as assessed in a small field trial was about 10%, nevertheless yield values were not provided on a ground area basis but per single plant. In a different context, Shavrukov *et al.* (2016) reported that a *TaDREB3* transgene transferred by conventional crossings to different genetic backgrounds of bread wheat improved drought resistance. However, as in the study discussed above (Yadav *et al.*, 2015), evaluation was performed in containers and yield was expressed per plant instead of per ground area.

## Improving photosynthesis to increase yields: both theoretically promising and difficult to achieve

Increasing leaf photosynthesis seems one of the obvious avenues for improving yield potential and resource use efficiency in a crop like wheat. About a decade ago (2008) several long-term projects that aimed to introduce C_4_ photosynthetic metabolism into C_3_ cereals were initiated (Hibberd *et al.*, 2008; von Caemmerer *et al.*, 2012), first with rice (http://www.C4Rice.com; last accessed 19/02/2019, funded by The Bill & Melinda Gates Foundation) and then in both wheat and rice (https://cordis.europa.eu/project/rcn/101753/factsheet/en; last accessed 19/02/2019; European Commission, among other projects). However, the optimistic predictions about producing C_4_ wheat and rice in the coming decades have been tempered as the full complexity of integrating C_4_ anatomy and metabolism within C_3_ plants has become clearer.

Other ways to increase leaf photosynthesis in wheat is by optimizing C_3_ metabolism. For example, the level of the Calvin–Benson cycle enzyme sedoheptulose-1,7-bisphosphatase (SBPase) has been increased through transformation and expression of a *Brachypodium distachyon* SBPase gene construct. Transgenic lines with increased SBPase protein levels and activity were grown under greenhouse conditions and showed enhanced leaf photosynthesis and increased total biomass and dry seed yield (Driever *et al.*, 2017). Although the increases in photosynthetic rates were moderate and expressed per unit area, total organ (i.e. leaf) or whole plant photosynthesis was not reported. Moreover, there is a need to account for negative interactions between the photosynthetic rate per unit area and leaf blade size and nitrogen accumulation.

Exploring natural variation in photosynthesis may also give clues as to the potential usefulness of this approach. The existing natural variation in photosynthetic capacity in a diverse panel of 64 elite wheat cultivars grown in the field in the UK was examined relative to biomass, yield and harvest index. Significant variations in photosynthetic capacity, biomass and yield were observed, although no consistent correlation was found between photosynthetic capacity of the flag leaf and grain yield when all cultivars were compared (Driever *et al.*, 2014). Furthermore, for the same set of genotypes, flag leaf longevity (stay green in terms of photosynthesis) and the duration of photosynthetic activity in the canopy can be further exploited to maximize grain filling (Carmo-Silva *et al.*, 2017). These studies indicate that rather than the actual rates of leaf photosynthesis on a per leaf area basis, what may be important is the active lifetime of these photosynthetic organs. On the other hand, the photosynthetic contribution of the ear and other non-laminar parts has been neglected, even though they may represent a key determinant of grain yield during the last part of the crop cycle (Sánchez-Bragado *et al.*, 2016). With their more stable and stress-resistant photosynthesis and extended stay green (Vicente *et al.*, 2018), these organs also have potential as a simpler system for introducing C_4_ metabolism (Hu *et al.*, 2018).

## Transgenic wheat for yield potential and stress resistance: chimera, panacea or somewhere in between?

To date, and despite the importance of wheat as a food and feed staple, no transgenic wheat of any nature has been approved for commercial cultivation. This is despite the fact that transgenic wheat cultivars could fulfil many objectives, including improving drought and other stress resistance (Box 2). Various factors, both economic and technical, may explain the lack of transgenic wheat. Significant investments in crop improvement have been unattractive for technology enterprises. As a self-pollinated crop, royalty collections from the sale of novel wheat varieties have historically faced difficulties due to farmer-saved seeds. In addition, drought-resistant transgenic crops may have lower returns compared to well-established transgenics and they might only make economic sense when they are combined with other transgenes (e.g. the gene for *Bacillus thuringiensis* toxin, Bt). This is well illustrated in maize where during the first three years of commercial exploitation in the US (which started in 2014), drought-resistant maize has generated a profit of only US$33 million, which represents a marginal (far less than one per thousand) proportion of the total profit generated by transgenic maize in the US (ISAAA, 2017). Moreover, unlike maize, wheat is polyploid, which means it is vital that genes identified as potential targets for yield selection be characterized for their interaction with other genes, while only certain allele combinations are beneficial for yield (Nadolska-Orczyk *et al.*, 2017). It has also been claimed that anti-GM consumer groups have boycotted any similar attempts to modify wheat, to the point where it was recently described as ‘*the cereal abandoned by GM*’ (Wulff and Dhugga, 2018). Nevertheless, this statement may only apply to Europe, rather than the rest of the world, and is clearly placed within the context of a propaganda war. In fact, drought-resistant transgenic wheat is a national research priority for countries such as China. In the case of Argentina, a wheat cultivar with an HB4 transgene is eventually close to approval (https://efarmnewsar.com/2018-11-16/will-argentina-be-the-first-country-approving-a-gmo-wheat.html; last accessed 19/02/2019) waiting for a political decision. Other drought-resistant crops, using HB4 technology, have already been approved. This is the position for Bioceres drought-resistant soybean, approved by the US Food and Drug Administration.

Box 2. **Publications covering transgenic crops**

Number of published articles with the following keywords in the title: (A) ‘transgenic maize/corn’, ‘transgenic wheat’ and ‘transgenic rice’; (B–D) proportion of results obtained from ‘transgenic rice’, ‘transgenic maize/corn’ and ‘transgenic wheat’, respectively, filtered by ‘photosynthesis’, ‘yield’, ‘drought’, ‘flooding’, ‘phosphorous’, ‘nitrogen’, ‘frost’, ‘salt’, ‘iron’, ‘zinc’, ‘Bt’, ‘disease’, ‘insect’ and ‘quality’. Information was obtained from Web of Science and included papers published since 1980 (the first report of successful transgenics in plants is Herrera-Estrella *et al.*, 1983).

Bt is the most common target in rice and maize. Drought and heat are important in all three cereals, comprising 15% of articles in transgenic maize/corn, 19% in rice and 24% of articles in transgenic wheat. ‘Salt’ is also common, comprising almost 29% of articles in wheat, 5% in maize and 17% in rice. Nevertheless, most articles published from 1980 to 2019 related to transgenics did not address (at least in the title) the major breeding objectives for the three crops, as shown by more than 50% of articles not falling into any of the categories related to photosynthesis, yield, abiotic stresses (drought, flooding, frost, salt), nutrients (phosphorus, iron, zinc), disease, Bt, insect or quality. This is a potential indication that the large number of articles not falling into the most common breeding targets are related to specific transcription factors, or genes that did not affect an overall process, mechanism or physiological response.

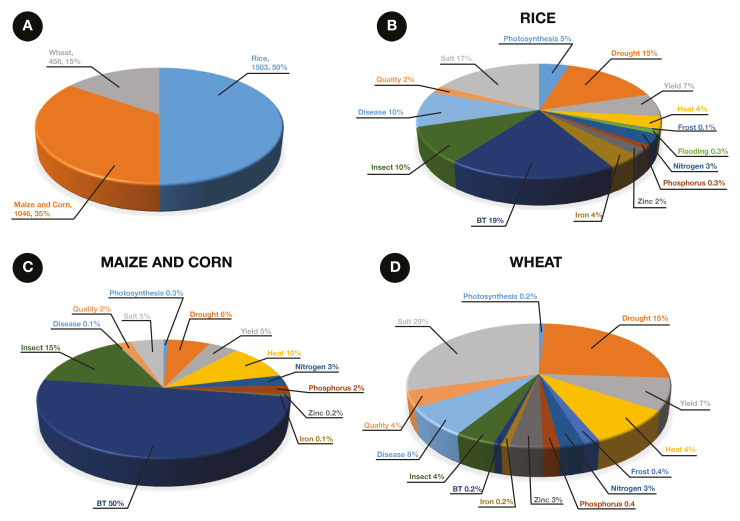

However, in the study by González *et al.* (2019), a selected transgenic line had a 6% larger yield and 9.4% greater water use efficiency than its control across evaluated environments, even if the comparative performance of the transgenic was higher under stress compared with non-stress conditions. In the case of the Bioceres soybean in Argentina, it is claimed to have had yield improvements of 13% on average during the past season, which was affected by a severe drought. In the Argentine pre-commercial transgenic wheat indicated in the previous paragraph, a huge (25%) increase in yield under stress conditions is claimed, but no independent data is available. The increase in productivity of the drought-resistant transgenic commercial maize has been reported to be less than 10% (Adee *et al.*, 2016). Therefore, to date the majority of reports conclude that yield increases using transgenics, even if significant, do not represent a ‘quantum leap’.

A limitation inherent to transgenics is the cultivar used for transformation, which after a decade or more following the initial transformation may have been superseded by modern higher-yielding varieties developed through conventional breeding. Thus, in the case of González *et al.* (2019), in the absence of drought the transformed wheat achieved grain yields similar to different commercial controls included in the experiments, which were comparatively modern local varieties expected to have improved adaptation as well as yield relative to the cultivar used as a basis for the transformation (Cadenza, released to the UK market in 1995). Even if the potential annual yield increase of wheat achieved as a result of conventional breeding is estimated at about 0.5% (Fischer *et al.*, 2011), after 10 years this process may generate a genetic advance in yield of 6% and therefore offset to a large extent (and at a much lower cost) the gains of the present successful transgenics. If we consider genetic gains of 1% as a result of conventional breeding (Lopes *et al.*, 2012), differences may be even less evident. In fact, a few years ago Hall and Richards (2013) concluded that the timescales required for major improvements in yield in farmer-ready cultivars through genetic engineering are likely to be measured in decades rather than years. Nevertheless, it is also worth mentioning that the bureaucracy related to transgenics approval contributes to making the process longer, which may cancel some of the benefits related to this technology when aimed at stress prone environments. Moreover, techniques such as gene system down-regulation via RNAi-based gene silencing, along with CRISPR/Cas gene editing, may accelerate the transformation-pipeline process in the future (Nadolska-Orczyk *et al.*, 2017).
